# Multi‐Soliton Microcombs Enable Ultrafast Nanometric‐Precision Ranging and Photon‐Level Detection

**DOI:** 10.1002/advs.202516806

**Published:** 2026-01-27

**Authors:** Jiawen Zhi, Xiaoyang Guo, Xusheng Yang, Brent E. Little, Sai T. Chu, Chenggang Shao, Mengyu Wang, Yan Liang, Peng Xie, Weiqiang Wang, Hanzhong Wu

**Affiliations:** ^1^ National Gravitation Laboratory MOE Key Laboratory of Fundamental Physical Quantities Measurement and School of Physics Huazhong University of Science and Technology Wuhan China; ^2^ State Key Laboratory of Transient Optics and Photonics Xi'an Institute of Optics and Precision Mechanics Chinese Academy of Sciences Xi'an China; ^3^ Department of Physics City University of Hong Kong Hong Kong China; ^4^ Jiangxi Provincial Key Laboratory of Opto‐Electronic Information Science and Technology Nanchang Hangkong University Nanchang China; ^5^ School of Optical‐Electrical and Computer Engineering University of Shanghai for Science and Technology Shanghai China; ^6^ Wangzhijiang Innovation Center for Laser Aerospace Laser Technology and System Department Shanghai Institute of Optics and Fine Mechanics Chinese Academy of Sciences Shanghai China; ^7^ Center of Materials Science and Optoelectronics Engineering University of Chinese Academy of Sciences Beijing China; ^8^ School of Electronic Information and Artificial Intelligence Shaanxi University of Science and Technology Xi'an China

**Keywords:** microcomb, multi‐soliton, nanometric‐precision, photon‐level detection, ultrafast ranging

## Abstract

Optical microcombs offer unprecedented capabilities in precision ranging due to their compact footprint, broad spectral bandwidth, and high repetition rates. However, practical deployment is limited by a fundamental compromise: single‐soliton states exhibit high coherence but low power conversion efficiency, whereas chaotic microcombs achieve higher efficiency at the expense of significant phase noise. Here, we overcome both limitations by implementing multi‐soliton microcombs in dual‐comb system. Multi‐soliton states provide higher efficiency and easier accessibility than single‐soliton states, while maintaining high coherence essential for nanometric precision compared to chaotic states. Experimental results indicate that utilizing three solitons in both combs, the measurement uncertainty is within ±17 nm and the precision reaches 1.43 nm at 2 µs and 3.42 pm at 500 µs. We also demonstrate vibration monitoring, spinning disk measurement, and unmanned aerial vehicle tracking. Moreover, with the five‐soliton signal comb and single‐soliton local comb, photon‐level ranging at femtowatt‐level power exhibits the uncertainty below ±9.5 µm, achieving the precision of 3.57 µm at 1 s and 202 nm at 50 s. Additionally, we conduct outdoor measurements at ∼270 m and non‐line‐of‐sight imaging. Multi‐soliton ranging outperforms single‐soliton approaches in precision, speed and efficiency, creating new opportunities in communications, spectroscopy, and optical time transfer.

## Graphical abstract

Our work first demonstrates dual‐multi‐soliton ranging and confirms its multifunctional capability. The method achieves ultrafast and nanometric‐precision measurement. For practical applications, we further extend the multi‐soliton states to photon‐level ranging. The proposed system surpasses the single‐soliton approaches in precision, speed, and efficiency, offering new opportunities for communications, spectroscopy, and optical time transfer.

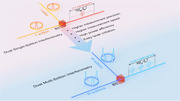

## 1 Introduction

Optical solitons in nonlinear cavities, sustained by the double balance between cavity dispersion and Kerr nonlinearity as well as cavity loss and parametric gain, have drawn considerable research interest in recent years. Theoretically described by the Lugiato–Lefever equation, such solitons constitute stable solutions existing on a continuous‐wave (CW) background [1]. Among implementations of soliton physics, Kerr microcombs [2, 3], represent one of the most significant advances, enabling diverse applications including optical communications [4, 5, 6, 7], time/frequency transfer [8, 9], precision spectroscopy [10, 11], and distance measurement [12, 13, 14] Length/distance is a fundamental physical quantity, and its precise measurement plays the vital role in fields such as light detection and ranging (LiDAR) [15], gravitational wave detection [16, 17], and 3D imaging [18]. Over the past two decades, frequency comb‐based ranging has advanced rapidly, with numerous methods being thoroughly developed and studied. These include intermode beat [19, 20], pulse cross correlation [21, 22, 23], pulse‐to‐pulse alignment [24, 25], dispersive interferometry [26, 27, 28, 29, 30], electro‐optic sampling [31, 32], and dual‐comb interferometry [33, 34, 35, 36, 37, 38, 39, 40, 41].

Recent advances in distance measurement have been significantly driven by the development of microcombs. In particular, the dissipative Kerr solitons (DKS) generated in microresonators have demonstrated considerable potential for high‐precision ranging. The formation dynamics of microcombs are now well established. Typically, the system transitions through a sequence of nonlinear states—such as the primary comb, secondary comb, modulation instability (chaotic state), and multi‐soliton states—before reaching the low‐noise single‐soliton state [42]. Owing to the high coherence and low phase noise, single soliton has been extensively employed in ranging. For instance, microcomb‐based pulse cross correlation enables arbitrary distance measurement with only a compact mechanical stage [43], improving mechanical stability and easing optical alignment. Dispersive interferometry using microcombs has also been implemented, effectively addressing dead‐zone limitations [44, 45]. Dual‐microcomb interferometry [46, 47, 48, 49, 50] supports ultrafast and precise measurements thanks to the large difference in the repetition rates. In addition, soliton microcombs allow multi‐channel frequency‐modulated continuous‐wave (FMCW) ranging [51, 52, 53], facilitating simultaneous distance and velocity measurements. It is notable that microcombs in these configurations generally operate in a free‐running manner, eliminating the need for complex active stabilization systems. Recently, rapid and precise distance measurement has also been performed using the hybrid comb system consisting of a fiber comb and a fully stabilized microcomb [54].

Nevertheless, the practical implementation of single‐soliton microcombs faces two main issues: the complexity of reliably accessing the single‐soliton state and its low power conversion efficiency from the CW pump [55]. Although soliton formation can be initiated by tuning the pump from the blue‐ to the red‐detuned regime, achieving a deterministic single‐soliton state requires additional precise control. More critically, the power conversion efficiency from the CW pump to the single‐soliton comb remains low, typically around ∼1%. Consequently, most of the optical power resides in the pump line, even though the pump itself constitutes one tooth of the comb spectrum. Very recently, chaos‐based unambiguous and parallel ranging in microresonators has been reported [56, 57], utilizing random amplitude and phase modulation across the chaotic comb spectrum. Compared to single soliton, chaotic states form more readily, reducing the need for intricate pump tuning. Furthermore, chaotic combs can achieve higher power conversion efficiencies, reaching levels of approximately ∼20%. However, due to their inherent phase incoherence, chaotic states cannot achieve sub‐wavelength precision in distance measurement. This presents a key challenge: how to simultaneously obtain high efficiency and high precision in soliton microcomb ranging.

In this work, we describe that multi‐soliton states in microresonators intrinsically enable ultrafast and precise distance measurement through dual‐multi‐soliton interferometry. This distinctive state has largely been studied theoretically and often viewed as impractical for precision measurement. In fact, however, the multi‐soliton state exhibits low phase noise—allowing nanometric precision compared to chaotic combs—while delivering higher optical power than the single‐soliton state, which improves the efficiency of CW‐to‐pulse conversion. It is also more readily accessible than the single‐soliton state. In dual‐multi‐soliton microcomb ranging, multiple interferograms are generated via multi‐pulse sampling within one update period, thereby enhancing both measurement precision and speed simultaneously. Our experimental results confirm these performance improvements.

## 2 Results

### 2.1 Measurement Concept of Dual‐Multi‐Soliton Interferometry

**FIGURE 1:**
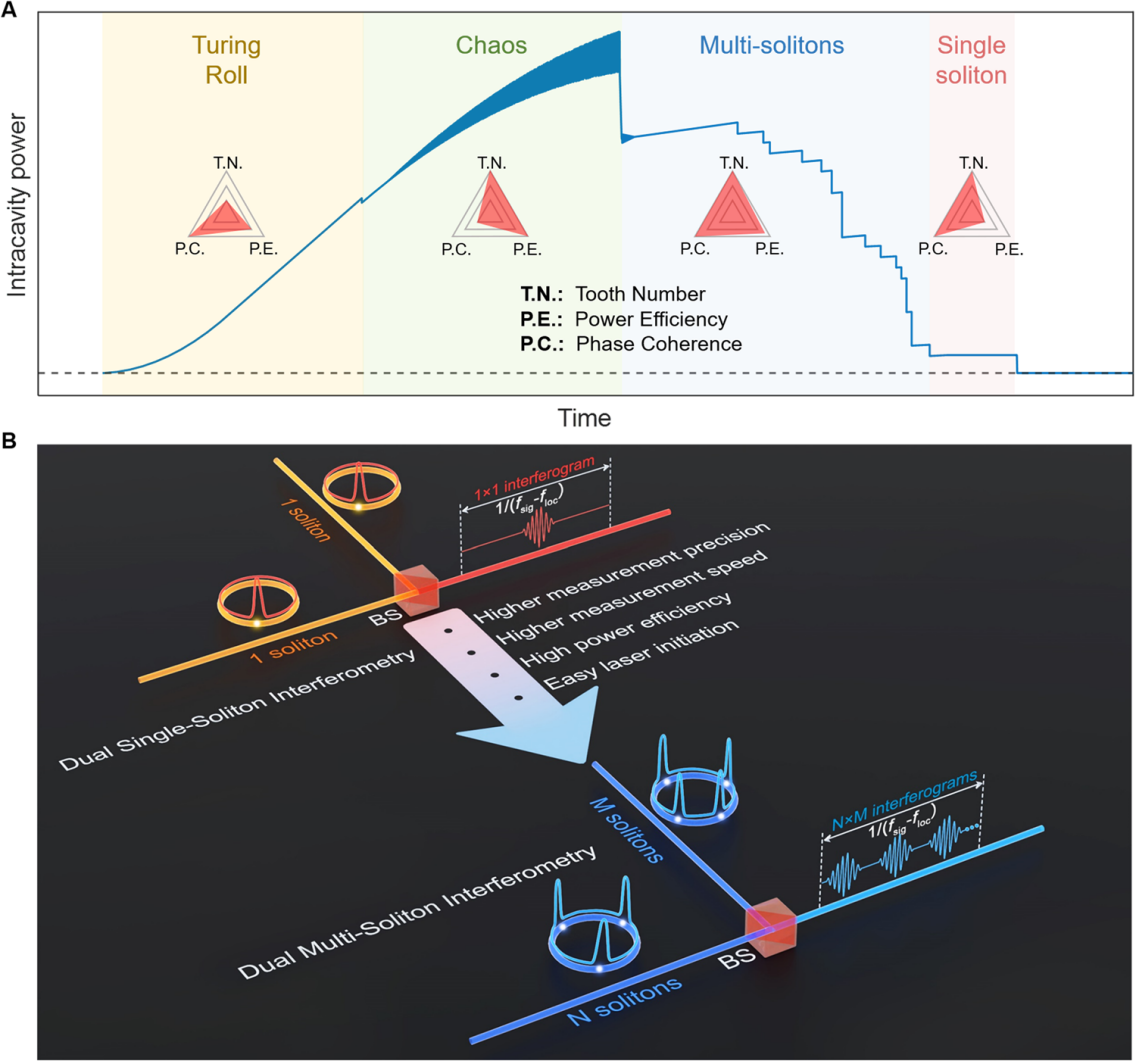
Measurement concept of dual‐multi‐soliton interferometry. A) Characteristics of different states in the microresonator, with consideration of ranging applications. Multi solitons exhibit excellent comprehensive performance, with sufficient tooth number, high power conversion efficiency, and high coherence. (B) Evolution from dual‐single‐soliton interferometry to dual‐multi‐soliton interferometry. In dual‐single‐soliton interferometry, one interferogram can be generated in one update period. In sharp contrast, *N*×*M* interferograms can be produced in one period for dual‐multi‐soliton interferometry, with *N* and *M* the soliton numbers of the signal and local combs, respectively. The measurement speed can be increased by *N*×*M* times, and moreover the precision can be enhanced by a factor of (*N*×*M*)^1/2^.

Figure 1 illustrates the motivation and measurement concept of our dual‐multi‐soliton distance meter. As depicted in Figure 1A, the formation of single soliton proceeds through a sequence of nonlinear states, including the primary/secondary combs, chaotic combs, and multi‐soliton states. While primary/secondary combs display superior coherence and moderate power conversion efficiency, their low comb‐line density fundamentally limits their utilization in ranging. Chaotic combs achieve remarkable power conversion efficiency, but the low coherence prevents high measurement precision. In contrast, single soliton in the microcavity provides high coherence across a broad spectrum, enabling high precision, yet it suffers from low power conversion efficiency. This inherent compromise between power efficiency and measurement precision has remained a fundamental challenge in microcomb‐based ranging. Multi‐soliton states resolve this limitation by achieving an optimal balance among power efficiency, measurement precision, and acquisition speed.

The comparison between dual‐single‐soliton interferometry and dual‐multi‐soliton interferometry is shown in Figure 1B. In the dual‐single‐soliton interferometry, only one interferogram is obtained per sampling period, i.e., 1/(*f_sig_*‐*f_loc_*), where *f_sig_* and *f_loc_* are the repetition rates of the signal and local combs, respectively. Thus, the target is measured only once per period. In contrast, dual‐multi‐soliton interferometry employs multiple low‐noise solitons in both combs, allowing the generation of much more interferograms within the same period. This significantly increases the number of measurements, accelerating the measurement speed by a factor of *N*×*M*, where *N* and *M* represent the soliton numbers in the signal and local combs, respectively. On the other hand, measurement precision can be quantified using the Allan deviation, defined as $\sigma_{y}\left(\tau \right)=\sqrt{\frac{1}{2(K-1)}\sum_{i=1}^{K-1}{\left(y_{i+1}-y_{i}\right)}^{2}}$, with *y_i_* being the *i*th of *K* fractional values average over the measurement interval *τ*. In dual‐multi‐soliton interferometry, *N*×*M* times measurement samples are acquired per period compared to the dual‐single‐soliton interferometry. Consequently, if the dominant noise is white noise, the Allan deviation can be reduced by (*N*×*M*)^1/2^ folds for the same averaging time. From the practical perspective, multi‐soliton states are more readily accessible, simplifying system operation by reducing the need for complex laser initiation routines.

### 2.2 Dual‐Multi‐Soliton Ranging System

**FIGURE 2:**
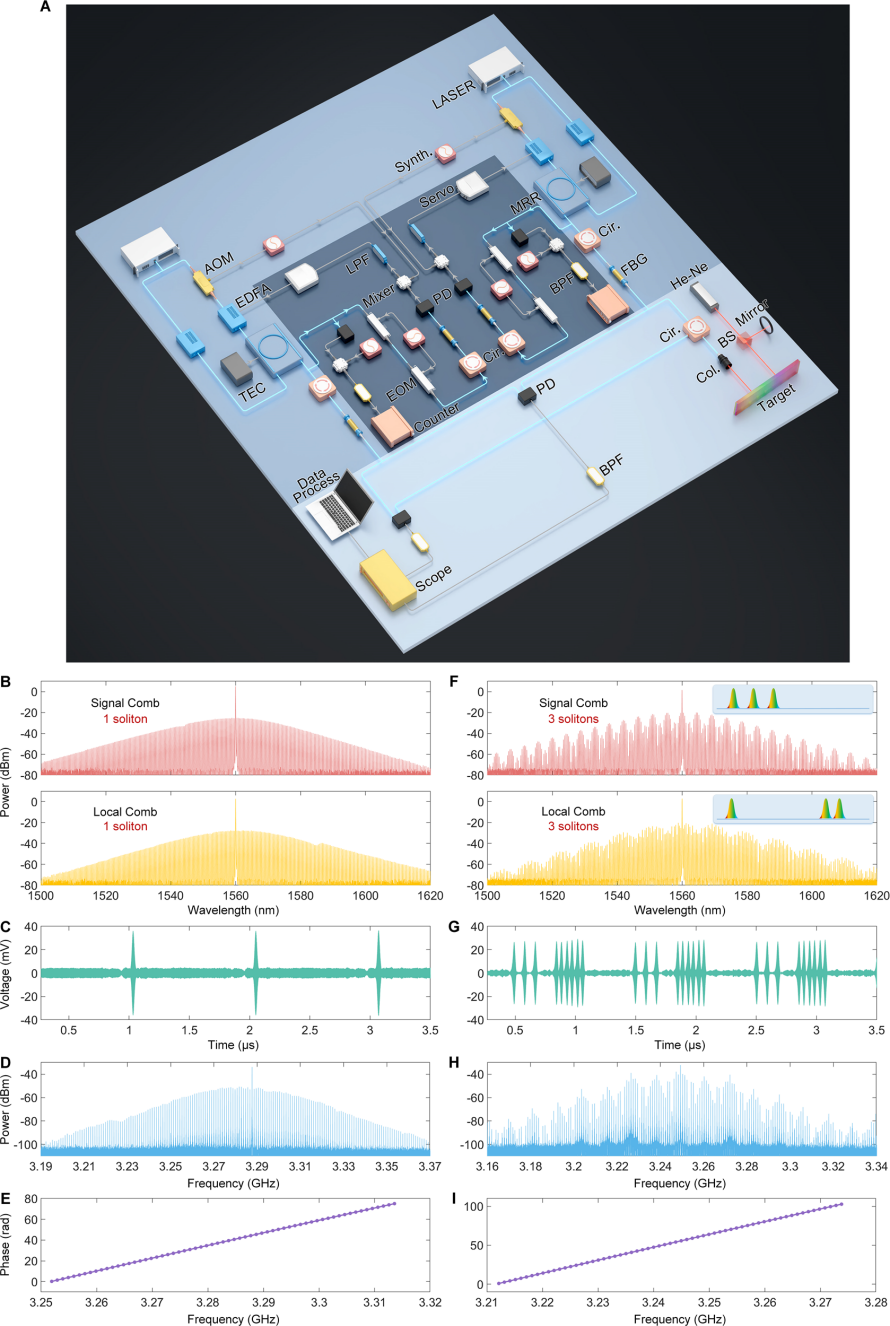
Experimental setup for dual‐microcomb ranging and time/frequency‐domain data. A) Experimental setup. AOM: acousto‐optic modulator; Synth.: signal synthesizer; EDFA: Er‐doped fiber amplifier; MRR: micro‐ring resonator; Cir.: circular; FBG: fiber Bragg grating; PD: photodetector; Col: collimator; BPF: band‐pass filter; TEC: temperature controller; BS: beam splitter. B) Spectrums of the signal and local combs, while both the combs are in the single‐soliton state. C) Dual‐comb interferograms. The update rate is 1 MHz, corresponding to the repetition rate difference. We see one interferogram in one period (1 µs). D) Electrical spectrum of the dual‐comb interferograms. The beat interval is 1 MHz, corresponding to the repetition rate difference. E) Unwrapped phase of the interferogram in (C), which is a straight line. F) Spectrums of the signal and local combs, while both the combs are in the multi‐soliton state, and with 3 solitons. The spectrums are not smooth, but with amplitude modulation due to the dispersive interferometry. G) Dual multi‐soliton comb interferograms with the update rate of 1 MHz. In sharp contrast to the results in (C), we see 9 (3 × 3) interferograms in one period. H) Electrical spectrum of the dual multi‐soliton comb interferograms. The beat interval is 1 MHz, corresponding to the repetition rate difference. We find the electrical spectrum is amplitude modulated. I) Unwrapped phase of one set of interferogram (one of the 9 interferograms), which is also a straight line.

Figure 2A depicts the experimental setup for the dual‐microcomb ranging. The multi‐soliton microcombs are generated based on the thermal balanced method, and more detailed information can be found in Ref. [48]. The power conversion efficiency varies between 4% and 28%, depending on the number of solitons. In our experiments, the signal comb has the repetition rate of 49.0 01117 GHz, while the local comb operates at 49.000117 GHz, yielding the repetition rate difference of 1 MHz. Both repetition rates are locked to the hydrogen maser, while the offset frequency remains free‐running. Please see Methods for the details. Within the dual‐comb ranging system, the signal comb is divided by a 50:50 coupler. One output serves as the reference beam, while the other is directed toward the target via an optical circulator as the measurement beam. Similarly, the local comb is split into two parts, which are separately combined with the reference and measurement beams to generate the reference and measurement signals, respectively. Fiber Bragg gratings are employed to suppress the CW background. The reference and measurement signals are detected by a pair of fast photodetectors and then sampled with the oscilloscope (LeCroy WaveMaster 820Zi) after passing through electrical band‐pass filters (3–3.4 GHz). The distance is determined through the following data process. The measured distance *L* is given by *L* = *ct*/(2 *n_g_*)×Δ*f*/*f_sig_*, where *c* is the light speed in vacuum, *t* is the real‐time delay, *n_g_* is the group refractive index of air, and Δ*f* is equal to *f_sig_*‐*f_loc_* ​. The delay *t* can be precisely extracted using either the Hilbert transform [58] or the fast Fourier transform [33, 37, 38] This constitutes the time‐of‐flight (TOF) measurement. In general, the performance of TOF can directly link to the phase of the optical carrier, which allows the distances to be resolved with sub‐wavelength precision [29, 33, 34]. Additionally, we demonstrate a simple method to extend the non‐ambiguity range by exploiting intrinsic microwave signals in the microresonator, and details are provided in the Supporting Information.

Compared with dual‐single‐soliton interferometry, dual‐multi‐soliton interferometry produces a greater number of interferograms. This increases the measurement speed and further enhances the precision. The direct comparison is presented in Figure 2. The optical spectrum of the single soliton exhibits the smooth *sech^2^* envelope corresponding to Figure 2B, whereas the spectrum of the three‐soliton state displays a modulation at specific frequencies, as depicted in Figure 2F. This modulation originates from dispersive interferometry among multiple pulses with different delays [59]. In the time domain, dual‐single‐soliton interferometry produces one interferogram per 1 µs period, while dual‐multi‐soliton interferometry generates nine interferograms (3×3) within the same period, as shown in Figure 2C,G. Therefore, *N*×*M* interferograms can be acquired per update period, where *N* and *M* denote the soliton numbers in the signal and local combs, respectively. Consequently, the measurement speed can be increased by *N*×*M* folds, and the precision can be improved by (*N*×*M*)^1/2^ at the same averaging time. Figure 2D displays the electrical spectrum of the dual‐single‐soliton interferometry, which also follows a *sech^2^* envelope. In contrast, the electrical spectrum of the dual‐three‐soliton interferometry reveals an envelope modulation that reflects the shape of the corresponding optical spectra. Please note that, the electrical spectrum is the same as that in Figure 2D, if only one set of interferograms (here, one in nine) is Fourier transformed. Figure 2E indicates the unwrapped phase of the interferogram in Figure 2C, obtained via Fourier transform.

**FIGURE 3:**
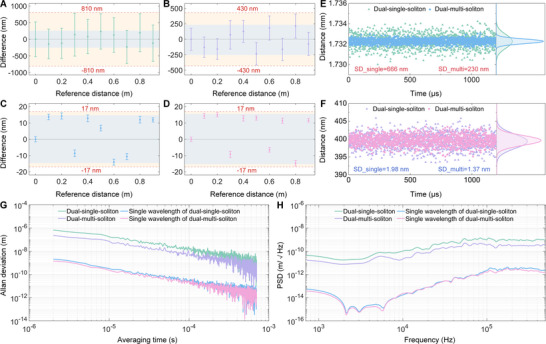
Measurement results of dual‐microcomb ranging. A) Results of the dual‐single‐soliton ranging based on the slope of the unwrapped phase. The purple‐shaded region indicates the measurement accuracy, reflecting the difference between the measured average and the reference value. The orange‐shaded region represents the measurement uncertainty. B) Results of the dual‐multi‐soliton ranging based on the slope of the unwrapped phase. C) Results of the dual‐single‐soliton ranging based on the phase of one single wavelength. D) Results of the dual‐multi‐soliton ranging based on the phase of one single wavelength. E) Measurement scatters for 1.2 ms of dual‐single‐soliton ranging and dual‐multi‐soliton ranging, when using the phase slope to determine the distances. The green triangles are the results of dual‐single‐soliton ranging, with the standard deviation of 666 nm, and the purple points correspond to the dual‐multi‐soliton ranging, with the standard deviation of 230 nm. F) Measurement scatters of dual‐single‐soliton ranging and dual‐multi‐soliton ranging, when using the phase of one single wavelength to measure the distances. Purple triangles represent dual‐single‐soliton ranging, with the standard deviation of 1.98 nm, while pink points correspond to dual‐multi‐soliton ranging, with the standard deviation of 1.37 nm. G) Allan deviations of different methods. The green line indicates the Allan deviation of dual‐single‐soliton ranging using the phase slope, and the purple line represents the Allan deviation of the dual‐multi‐soliton ranging using the phase slope. The blue line shows the Allan deviation of dual‐single‐soliton ranging using the phase of one wavelength, and the pink line represents the Allan deviation of the dual‐multi‐soliton ranging using the phase of one wavelength. H) Linear spectral densities of different methods. The green line indicates the PSD of dual‐single‐soliton ranging using the phase slope, and the purple line represents the PSD of the dual‐multi‐soliton ranging using the phase slope. The blue line shows the PSD of dual‐single‐soliton ranging using the phase of one wavelength, and the pink line represents the PSD of the dual‐multi‐soliton ranging using the phase of one wavelength.

We first evaluate the measurement performance using the linear translation stage and the results are presented in Figure 3. Due to the limited laboratory space, the measured distances are confined to 1 m range with 0.1 m step size. An incremental distance meter (Renishaw XL80) is employed to evaluate the performance of our comb distance meter. The air refractive index is corrected according to the Ciddor formula [60]. The acquired interferograms are Fourier‐transformed, and distances are extracted from the slope of the unwrapped phase via the time‐of‐flight method. Furthermore, by utilizing the phase of the single wavelength, the measurement uncertainty can be improved to within one wavelength.

Figure 3A presents the ranging results obtained with dual‐single‐soliton interferometry using the phase‐slope method. Each midpoint represents the average of 20 consecutive measurements, with the measuring time of 1 µs per measurement. The errorbars indicate the standard deviation corresponding to 1 µs averaging time. Over the measured 1 m range, the uncertainty remains below ±810 nm. We then employ the single wavelength (1560.018 nm) to finely measure the distances. As indicated in Figure 3C, the uncertainty improves to within ±17 nm, corresponding to a relative value of 1.7 × 10^−^^8^. A long‐term measurement of approximately 1.2 ms is performed at 1 m position. The measurement scatters obtained via the phase‐slope method are shown in Figure 3E, where the green triangles represent the dual‐single‐soliton ranging results, with a standard deviation of 666 nm. The scatters resulting from the single wavelength are depicted in Figure 3F using purple triangles, and the standard deviation is 1.98 nm. As illustrated in Figure 3G, the Allan deviations reach 675 nm at 2 µs and 1.5 nm at 500 µs averaging time for the phase‐slope method. When using the single‐wavelength phase, the Allan deviations achieve 2.12 nm at 2 µs and 5.48 pm at 500 µs averaging time.

We next conduct distance measurements using dual‐multi‐soliton interferometry (here, with 3×3 solitons). The increased number of interferograms acquired per period enables higher measurement speed and improved precision. As shown in Figure 3B, the measurement uncertainty is less than ±430 nm over 1 m range. Compared with the dual‐single‐soliton results in Figure 3A, the standard deviations are improved by approximately 2.8 folds, consistent with the 9 times increase in measurements per period. When the single wavelength is used, the measurement uncertainty is also below ±17 nm. No significant improvement relative to the dual‐single‐soliton ranging is observed at this level, which could be attributed to the system approaching its tens‐of‐nanometers measurement limit. The measurement scatters derived from the phase‐slope method is presented in Figure 3E, where purple points correspond to the dual‐multi‐soliton measurements. The associated standard deviation is 230 nm, representing 2.89 folds improvement over the dual‐single‐soliton result. In Figure 3F, the scatters for the single‐wavelength method is shown using pink points, with the standard deviation of 1.37 nm. For the phase‐slope method, the Allan deviations are 233 nm at 2 µs and 1.04 nm at 500 µs averaging time, as indicated in Figure 3G. When the single‐wavelength phase is employed, the Allan deviations achieve 1.43 nm at 2 µs and 3.42 pm at 500 µs averaging time.

The measurement sensitivity is evaluated using the power spectral density (PSD), with results presented in Figure 3H. When using the phase‐slope method, the PSD reaches 17.9 pm/Hz^1/2^ at 2 kHz in the dual‐single‐soliton measurements and improves to 7.53 pm/Hz^1/2^ at 2 kHz for dual‐multi‐soliton measurements. For the single‐wavelength approach, the PSD improves by approximately two orders of magnitude, remaining below 1 pm/Hz^1/2^ across the 1–70 kHz band. This demonstrates that our system can resolve displacements at the picometer level. Overall, the results confirm that dual‐multi‐soliton ranging exhibits superior performance compared to the dual‐single‐soliton ranging.

### 2.3 Dynamic and Long‐Distance Measurements

**FIGURE 4:**
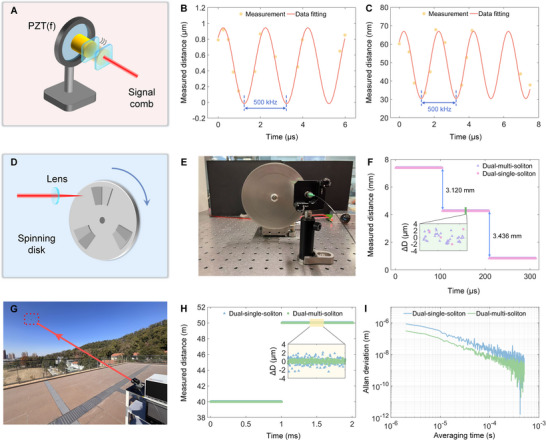
Schematic and results of dynamic and long‐distance measurements. A) Schematic of the high‐frequency vibration measurement based on a piezoelectric transducer. B) Results of the vibration measurement. The amplitude is 961 nm, and the vibrating frequency is 500 kHz. C) Results of the vibration measurement. The amplitude is 36.5 nm, and the vibrating frequency is 500 kHz. The yellow points shows the measurement samples, and the red line is the fitted curve. D) Schematic of the spinning disk measurement. E) Photograph of the spinning disk measurement. F) Results of the spinning disk measurement. The enlarged view shows that more samples can be obtained by using multi‐soliton interferometry. G) Photograph of long distance measurement out of lab. H) Results of long distance measurement, at about 40 m and 50 m distances. The inset indicates that the scatters of the dual‐multi‐soliton ranging is more stable. I) Allan deviations at 50 m distance. The precision can be improved by using dual‐multi‐soliton ranging.

The large repetition‐rate difference of the microcombs enables high‐speed acquisition, making the system suitable for dynamic measurements. We perform a series of dynamic tests to examine its fast‐measurement capability. Figure 4A illustrates the schematic of vibration measurement setup, while the target mirror is attached to a piezoelectric transducer (PZT) with a bandwidth in the kHz range. To highlight the dynamic performance of dual‐multi‐soliton ranging, the experimental parameters are adjusted as follows: the repetition‐rate difference between the signal and local combs is set to 174 kHz, while the PZT is driven at 500 kHz. Under these conditions, the Nyquist law is not satisfied. Nevertheless, by employing dual‐multi‐soliton interferometry, a larger number of samples can be acquired owing to the increased measurement speed. Here, the soliton numbers of the signal (*N*) and local (*M*) combs are 10 and 1, respectively. This configuration yields 10 interferograms per update period, enabling the capture of ultrafast dynamics. The measurement results are presented in Figure 4B,C. The vibrations with amplitudes of 961 and 36.5 nm, both at 500 kHz, are clearly resolved. This shows that our system can monitor the high‐frequency, nanometer‐scale vibrations even when the repetition‐rate difference Δ*f* does not meet the requirement of Nyquist law. Please note that, increasing the repetition rate difference Δ*f* can also enhance the measurement speed (e.g., several hundreds of MHz, in this case the following electrical circuits should be sufficiently fast so as to sample the resulting signals), and on this basis the update speed in our work is *N*×*M*×Δ*f* (larger than Δ*f*).

Subsequently, we measure a rapidly spinning disk with steps of different heights (here, *N* = 9, and *M* = 1), as depicted in Figure 4D, and the corresponding photograph is shown in Figure 4E. The disk has the radius of about 15 cm and rotates at 10000 rounds per minute, resulting in the linear speed at its edge of about 160 m/s. The measurement results are indicated in Figure 4F, and the different heights of approximately 3.120 and 3.436 mm can be clearly captured. Furthermore, the enlarged view presents that the dual‐multi‐soliton ranging acquires a higher sampling density compared to the dual‐single‐soliton approach.

To assess long‐distance measurement performance, we conduct ranging experiments on the unmanned aerial vehicle (UAV) equipped with a corner cube (here, *N* = 3, *M* = 3). The photograph taken during the rooftop experiment is provided in Figure 4G. Figure 4H displays the measured positions when the UAV is located approximately 40 m and 50 m from the controller, respectively. The scatters obtained using dual‐multi‐soliton ranging are more stable than those from the dual‐single‐soliton method. Notably, the high frame rate displayed in Figure 4H essentially reflects the measurement speed of the fractional distance. In the long‐time measurements at the distance of about 50 m, the Allan deviations for dual‐single‐soliton ranging are 852 nm at 2 µs and 5 nm at 500 µs averaging time. For dual‐multi‐soliton ranging, the corresponding Allan deviations are 308 nm at 2 µs and 2.06 nm at 500 µs averaging time, as illustrated in Figure 4I.

### 2.4 Photon‐Level Dual‐Microcomb Ranging

**FIGURE 5:**
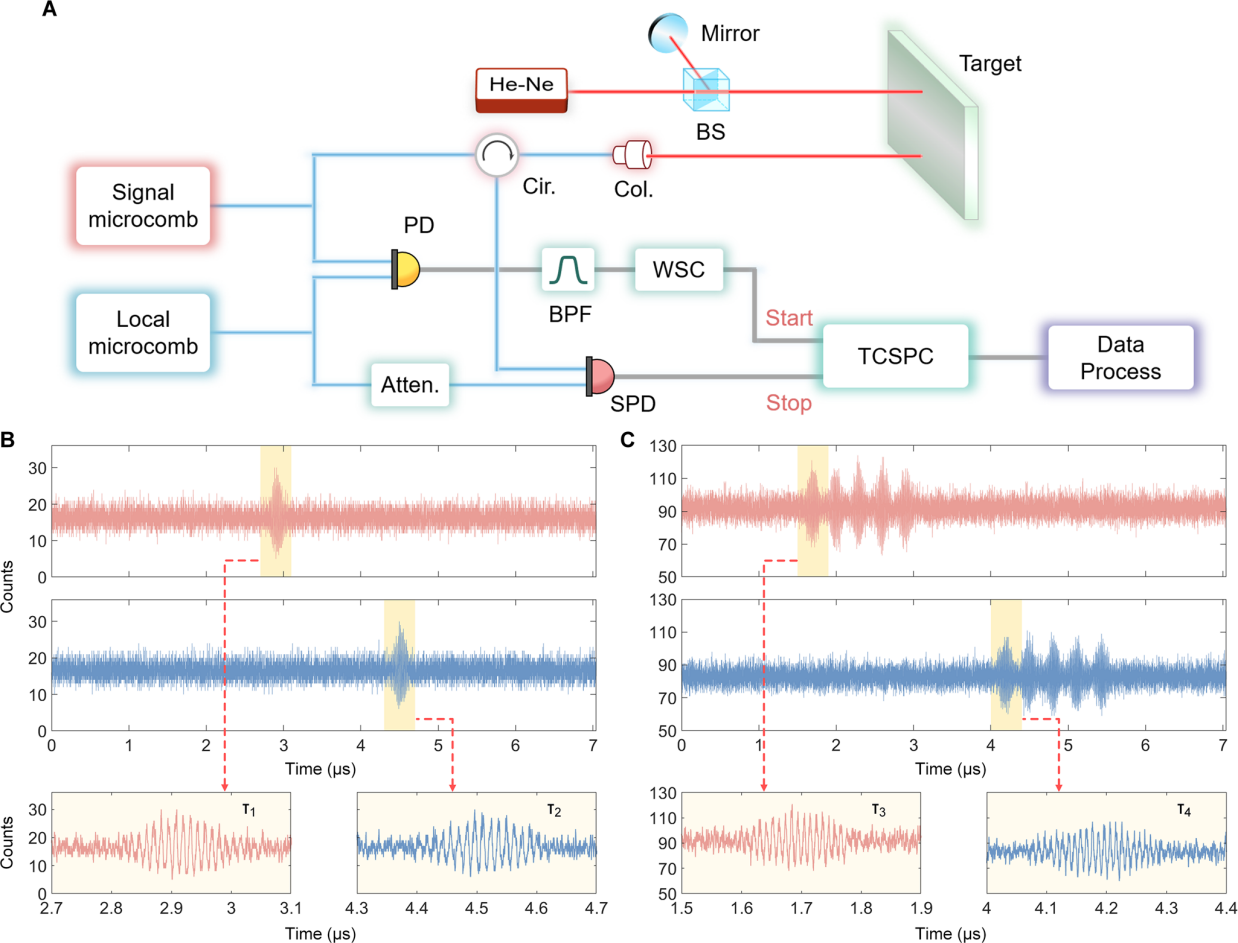
Experimental setup for photon‐level dual‐microcomb ranging and interferograms. A) Experimental setup. Cir.: circular; PD: photodetector; Col.: collimator; Atten.: power attenuator; BPF: band‐pass filter; WSC: wave shaping circuit; SPD: single photon detector; TCSPC: time‐correlated single‐photon counter; BS: beam splitter. B) Interferograms measured by the TCSPC at different distances, with dual‐single‐soliton configuration. We find good fringes at photon level, which can be used to determine the distances. C) Interferograms measured by the TCSPC at different distances, with dual‐multi‐soliton configuration (here, *N* = 5, and *M* = 1). Since we have more solitons here, the photon number is accordingly increased.

Photon‐level ranging is highly attractive for numerous applications, including lunar laser ranging [61] and non‐line‐of‐sight imaging [62] etc. Single‐photon dual‐comb ranging [63] not only relaxes the requirement on returned optical power but also preserves high precision. Here, by employing the single‐photon detector (SPD), we further extend the concept to photon‐level dual‐multi‐soliton ranging. The comparative analysis of its performance relative to the dual‐single‐soliton method is conducted, and the updated experimental setup is illustrated in Figure 5A. The tailored circuit reshapes the reference signal into a square wave, which then serves the start input of the time‐correlated single‐photon counter (TCSPC. PicoQuant MultiHarp 150). In our experiments, this circuit is required to successfully observe the interferograms. The measurement signal is heavily attenuated by the fiber attenuators and subsequently detected by a home‐made gated InGaAs SPD. The SPD output is connected to the stop input of the TCSPC, allowing interferograms to be acquired at the photon level.

Figure 5B displays a pair of interferograms corresponding to distinct distances, measured using the photon‐level distance meter in the dual‐single‐soliton configuration. One interferogram is observed per measurement period. The repetition rate difference is locked at 141 kHz, resulting in the time window of 7.09 µs. The integration time of the photon counter is set to 1 s (i.e., the measuring time for a single measurement), and the total detected photon count is approximately 7 × 10^5^, equivalent to the optical power of about 93 fW. When the target mirror is displaced approximately 0.7 mm away, the interferogram shifts accordingly. Subsequently, the soliton numbers are adjusted to 1 in the local comb and 5 in the signal comb. As shown in Figure 5C, this configuration yields five interferograms within a single period, each of which can be Fourier‐transformed to determine distance. The increased number of solitons raises the total photon count to 3.7 × 10^6^, corresponding to the optical power of 472 fW.

**FIGURE 6:**
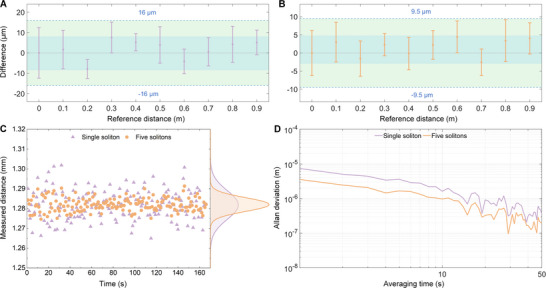
Measurement results of photon‐level dual‐microcomb ranging. A) Results of the photon‐level dual‐single‐soliton ranging. The blue‐shaded region indicates the measurement accuracy, reflecting the difference between the measured average and the reference value. The green‐shaded region represents the measurement uncertainty. B) Results of the photon‐level dual‐multi‐soliton ranging. The midpoints show the average of 10 measurements, and the error bar indicates the standard deviation. C) Measurement scatters of the dual‐single‐soliton ranging and dual‐multi‐soliton ranging, for 160 s measurement. The purple triangles shows the results of dual‐single‐soliton ranging, and the orange points correspond to the dual‐multi‐soliton ranging. Dual‐multi‐soliton ranging can result in more stable results. D) Allan deviation of the dual‐single‐soliton ranging and dual‐multi‐soliton ranging. The measurement precision can be improved by using dual‐multi‐soliton ranging.

Figure 6A presents the measurement results of the dual‐single‐soliton ranging at the photon level, and the measurement uncertainty is within ±16 µm. The midpoints show the average of 10 measurements, and the error bars indicate the standard deviation. By contrast, when the number of interferograms per period is increased to five, the uncertainty improves to less than ±9.5 µm over 1 m range, as shown in Figure 6B. To assess the long‐term stability, we conduct prolonged measurements, and the scatters are displayed in Figure 6C. The standard deviations are found to be 7.22 µm for dual‐single‐soliton ranging and 3.44 µm for dual‐multi‐soliton ranging. The corresponding Allan deviations are shown in Figure 6D. For dual‐single‐soliton ranging, the Allan deviations achieve 7.39 µm at 1 s and 364 nm at 50 s. In the case of dual‐multi‐soliton ranging, the values improve to 3.57 µm at 1 s and 202 nm at 50 s averaging time. These results demonstrate that the dual‐multi‐soliton approach enhances the measurement precision by nearly a factor of $\sqrt{5}$.

**FIGURE 7:**
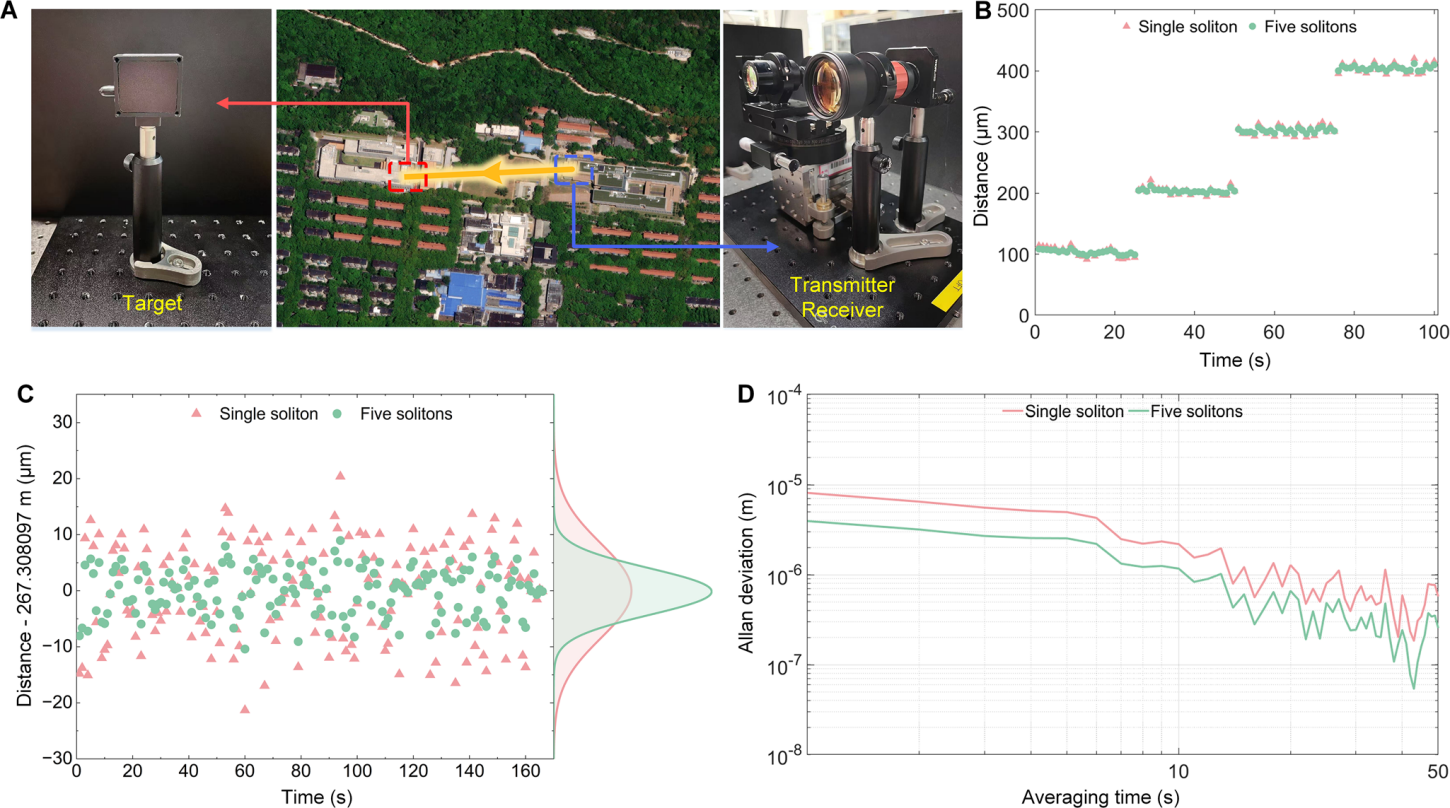
Schematic of the photon‐level ranging at long range and measurement results. A) Ranging schematic. B) Displacement measurement results with 100 µm step size. The red triangles show the results of dual‐single‐soliton ranging, and the green points indicate the results of the dual‐multi‐soliton ranging. C) Measurement scatters of the dual‐single‐soliton ranging and dual‐multi‐soliton ranging, for 160 s measurement. The red triangles show the results of dual‐single‐soliton ranging, and the green points correspond to the dual‐multi‐soliton ranging. D) Allan deviation of the dual‐single‐soliton ranging and dual‐multi‐soliton ranging, at 267 m distance.

High‐precision photon‐level ranging supports diverse practical applications. In this subsection, we perform its capability by measuring distances to an arbitrary target outside the laboratory. As illustrated in Figure 7A, the transmitter and receiver are placed on the laboratory rooftop in the off‐axis configuration, with the target positioned on another building approximately 267 m away. The transmitted beam has the diameter of 14 mm and a divergence angle of 0.14 mrad. The off‐axis optical system collects the returned signal. The target is moved in steps of 100 µm, and the resulting small displacements are clearly resolved, as shown in Figure 7B. The scatters are presented in Figure 7C. The calculated standard deviations are 8.43 µm for dual‐single‐soliton ranging and 4.15 µm for dual‐multi‐soliton ranging. Figure 7D displays the Allan deviations for both methods. The dual‐single‐soliton ranging achieves the Allan deviations of 8.11 µm at 1 s and 573 nm at 50 s. In comparison, the dual‐multi‐soliton ranging improves these values to 3.95 µm at 1 s and 256 nm at 50 s averaging time.

### 2.5 Non‐Line‐of‐Sight Imaging

**FIGURE 8:**
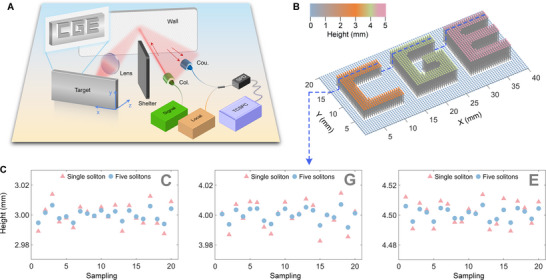
Schematic of the non‐line‐of‐sight imaging by dual‐multi‐soliton ranging and measurement results. A) Measurement schematic. Col.: collimator; Cou.: coupler. B) 3D imaging of the sample with three letters of different heights. C) Measurement results of one line on the sample.

In this subsection, we demonstrate the non‐line‐of‐sight (NLOS) imaging utilizing dual‐comb ranging. The schematic of the experimental setup is illustrated in Figure 8A. The signal microcomb is output through a collimator onto the relay wall. The scattered light from the wall is then focused by a lens onto the sample featuring the letters “C,” “G,” and “E” to extract their depth (z‐axis) information. These letters are designed with distinct heights. The sample is mounted on a 2D translation stage, which is stepped in 0.5 mm increments to acquire the in‐plane (x‐axis and y‐axis) spatial information. Light returning from the sample is again scattered by the wall and subsequently collected via a fiber coupler. Finally, the collected photons are then interfered with the local microcomb to generate the interferograms. The reconstructed 3D image, presented in Figure 8B, clearly reveals the three letters with respective heights of 3, 4, and 4.5 mm. Figure 8C displays the representative line profile extracted from the sample, highlighting the height variation. Throughout the measurements, the dual‐multi‐soliton ranging exhibits superior stability compared to the dual‐single‐soliton approach.

## 3 Discussion

While dual‐single‐soliton ranging supports ultrafast and precise distance measurements, the single‐soliton state in the microcavity suffers from lower power‐conversion efficiency and is less readily accessible. In contrast, multi‐soliton states in the microresonators provide higher power‐conversion efficiency, are more readily attainable, and preserve essential low‐noise properties. This combination makes them suitable for various precision measurements. This work focuses on precise distance measurement, where the measurement uncertainty arises from several factors: the single‐wavelength uncertainty, the correction of the air refractive index, the data‐processing algorithms, and the environmental stability. The repetition rate of the microcomb is locked to the Hydrogen maser with a stability of 10^−^^13^ at 1 s. Although the pump laser (i.e., the offset frequency) is not actively stabilized, its stability remains at the level of 10^−^^9^ at 1 s [57], with even better short‐term performance. The contribution of the air refractive index is significantly suppressed in our system for two reasons. First, the comb distance meter and the reference distance meter share the same environmental conditions. Second, the measurement speed is sufficiently high that the air can be considered stable over the short acquisition time. Similarly, environmental disturbances such as vibration and air turbulence contribute to the measurement uncertainty, yet rapid measurement helps mitigate their impact, thereby improving precision. It is important to point out that multi‐soliton interferometry generates much more interferograms per period than single‐soliton interferometry, without requiring additional delay lines [64]. This directly increases the number of measurement samples within each period. Consequently, both the measurement speed and precision are enhanced simultaneously, owing to the inherently low noise of multi‐soliton states. It is important to note that specifically, the high repetition rate (∼50 GHz) limits the non‐ambiguity range to approximately 3 mm. Therefore, two separate measurements (with larger and smaller non‐ambiguity ranges, respectively) are needed to determine the long distances coarsely and finely. Please see the details of the non‐ambiguity range expansion in Supporting Information.

Although dual‐microcomb ranging can achieve precise distance measurement even with the free‐running comb lasers, we recommend locking the repetition rate to maintain measurement precision. When the repetition rate is completely free‐running, the interferograms exhibit a continuous breathing motion in our experiments, caused by slight drifts in the repetition‐rate difference. This issue becomes particularly critical in the single‐photon ranging, where stringent and high‐quality synchronization between the start and stop signals of the TCSPC is essential. It should also be noted that the dark count of the TCSPC can make a contribution to the measurement uncertainty, resulting in the noisy interferograms, which can be estimated by the standard deviation. Furthermore, sufficient integration time (i.e., ∼1 s) is required in single‐photon detection to accumulate enough photon counts for clear interferogram observation. To reduce the measurement time, we consider that the fragmentary interferograms could also be utilized to extract the distance information.

## 4 Conclusions

In this work, we present the first demonstration of dual‐multi‐soliton ranging and explore its multifunctional applications. Previously, the coherent multi‐soliton state in the microresonator is often considered impractical. Compared with the single‐soliton state, multi solitons carry higher optical power and inherently offer improved pump‐to‐comb conversion efficiency. Simultaneously, multi‐soliton generation is considerably more straightforward to achieve than single‐soliton formation. Furthermore, multi solitons exhibit low phase noise, which makes them particularly suited for precision measurement.

Our experimental results confirm that dual‐multi‐soliton interferometry generates more interferograms than the dual‐single‐soliton interferometry. This increased sampling improves the measurement precision by the factor of (*N*×*M*)^1/2^ and correspondingly refines the measurement uncertainty. The results show that the measurement uncertainty is well within ±17 nm, and the unprecedented precision achieves 1.43 nm at 2 µs and 3.42 pm at 500 µs averaging time. Equally important, the measurement speed is also enhanced by *N*×*M* folds. This advancement enables ultrafast dynamic measurement beyond Nyquist law constraints and overcomes the fundamental speed limitations imposed by the repetition‐rate difference. To bridge the gap between laboratory research and field applications, we further demonstrate the photon‐level dual‐multi‐soliton ranging. Despite operating at tens of femtowatt optical power, the system achieves the measurement uncertainty below ±9.5 µm, with the precision of 3.57 µm at 1 s and 202 nm at 50 s. In addition, the long‐distance outdoor measurements over 267 m successfully resolve 100 µm steps. Finally, we implement the non‐line‐of‐sight imaging. These results collectively validate the superior performance of dual‐multi‐soliton ranging over dual‐single‐soliton ranging. Please note that the soliton number can be further increased. Nonetheless, more interferograms can be generated, and temporal overlap will occur, which should be resolved in the future. We anticipate that multi solitons in the microcavity can be also exploited in numerous other applications, including absorption spectroscopy [10, 11], high‐capacity communications [4, 5], and optical time transfer [8, 9], In absorption spectroscopy, the increased number of pulses in a multi‐soliton microcomb can enhance the sampling rate with the target gas. Through repeated averaging over these additional optical samples, the signal‐to‐noise ratio would be improved, leading to higher detection sensitivity and more reliable identification of weak absorption lines. For optical communications, the multi‐soliton state can offer more optical pulses per repetition period. This characteristic makes it a promising candidate for supporting higher‐capacity optical communications. In time transfer, multi‐soliton streams provide a greater number of coherent temporal markers. These additional markers enable finer resolution in timing comparison and improve the stability of long‐distance dissemination. Our work could open a new avenue for the future multi‐soliton science and technology.

## 5 Methods

### 5.1 Stabilization of the Repetition Rate

**FIGURE 9:**
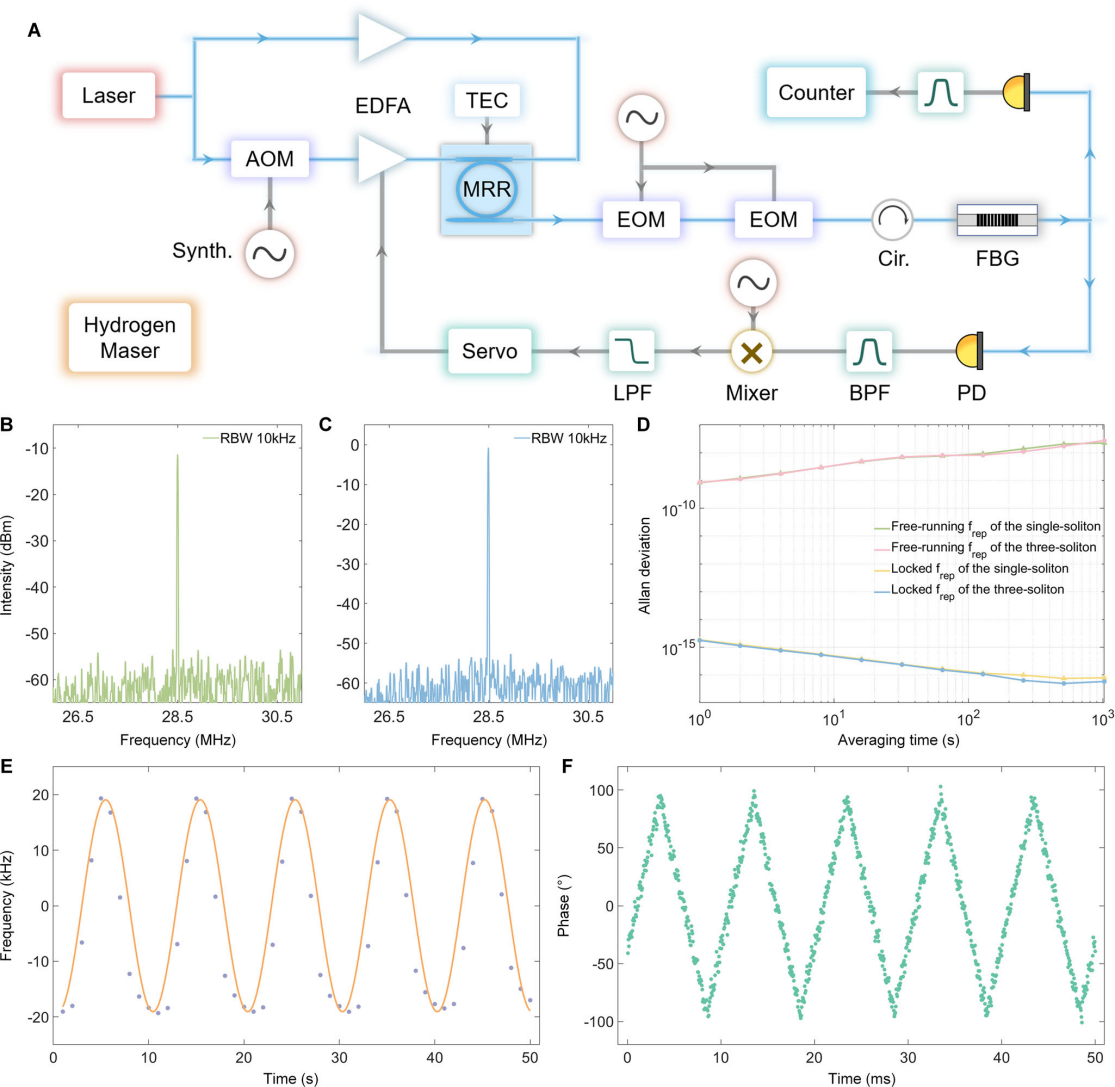
Locking schematic of repetition rate and performance evaluation results. A) Experimental setup. AOM: acousto‐optic modulator; Synth.: signal synthesizer; EDFA: Er‐doped fiber amplifier; MRR: micro‐ring resonator; Cir.: circular; FBG: fiber Bragg grating; PD: photodetector; BPF: band‐pass filter; LPF: low‐pass filter; TEC: temperature controller; EOM: electro‐optic modulator. B) Beat note after inserting multi side bands using EOMs, with 42 dB signal‐to‐noise ratio, in the single‐soliton *f_rep_* locking. C) Beat note after inserting multi side bands using EOMs, with 52 dB signal‐to‐noise ratio, in the three‐soliton *f_rep_* locking. D) Allan deviations of the repetition rate with different soliton numbers. E) Results of repetition rate modulation, with 10 s period and about 40 kHz tuning range. F) Results of the soliton phase modulation, with 100 Hz modulation frequency and about 200 degrees tuning range.

In our experiments, the repetition rates of the signal and local combs are phase‐locked to the Hydrogen maser (iMaser3000). Figure 9A depicts the locking scheme, which follows the same configuration shown in Figure 2. Two electro‐optic modulators (iXblue MPZ‐LN‐10) are employed to generate multiple sidebands between adjacent comb modes [65], producing a low‐frequency beat note *f_b_* given by *f_b_* = *f_rep_*‐*K*×*f_EOM_*. Here, *K* is an integer (set to 4 in this work) and *f_EOM_* denotes the EOM drive frequency (12.242 970 GHz, stabilized to the Hydrogen maser). It is worth noting that all signal generators and measurement instruments in our setup are referenced to the hydrogen maser. Before detecting *f_b_*, the optical circulator and the fiber Bragg grating are used to greatly reduce the central pump power, thereby preventing photodetector saturation. The resulting *f_b_* appears at approximately 28.5 MHz, as shown in Figure 9B, with the signal‐to‐noise ratio exceeding 42 dB. A standard phase‐locked loop is implemented to stabilize the repetition rate by feedback controlling the power of the auxiliary pump. With the soliton number increased to 3, the corresponding beat signal *f_b_* displays both higher power and higher signal‐to‐noise ratio of 52 dB, as depicted in Figure 9C.

The locking performance is indicated in Figure 9D. Under the free‐running condition, the *f_rep_* stability of both the single‐soliton and three‐soliton states is nearly identical, with Allan deviations of 8.28 × 10^−^^10^ at 1 s, 9.16 × 10^−^^9^ at 128 s, and 2.24 × 10^−^^8^ at 1024  s averaging time. When the phase‐locked loop is activated, the stability closely follows that of the hydrogen maser. For the single‐soliton state, the Allan deviations are 1.85 × 10^−^^15^ at 1 s, 1.15 × 10^−^^16^ at 128 s, and 7.93 × 10^−^^17^ at 1024 s averaging time. With three solitons, the corresponding values are 1.77 × 10^−^^15^ at 1 s, 1.08 × 10^−^^16^ at 128, and 5.83 × 10^−^^17^ at averaging time. We further modulate the repetition rate by applying a sine wave to the reference signal. As shown in Figure 9E, the repetition rate is precisely changed by ±20 kHz over 10 s period, which is valuable for the extending of the non‐ambiguity range. Separately, a triangle‐wave modulation is employed on the reference phase to control the soliton phase (i.e., the soliton position in microresonator). Figure 9F confirms that the soliton phase can be continuously adjusted over ±100 degrees under 100 Hz modulation frequency.

## Author Contributions

J.Z. and H.W. conceived the dual‐multi‐soliton interferometry technique. J.Z., X.Y., Y.L., and H.W. carried out the measurement. J.Z., X.G., M.W., Y. L., C.S., and H.W. analyzed the data. J.Z., X.G., W.W., and H.W. wrote the manuscript. B.E.L., S.T.C., P.X., and W.W. designed and fabricated the microresonator. P.X., W.W., and H.W. supervised the research work.

## Funding

This work is supported by the National Key Research and Development Program of China no.2022YFC2204601; National Natural Science Foundation of China nos. 12275093 and 62175152.

## Conflicts of Interest

The authors declare no conflicts of interest.

## Supporting information




**Supporting File**: advs74073‐sup‐0001‐SuppMat.docx.

## Data Availability

The data that support the findings of this study are available from the corresponding author upon reasonable request.
