# Supplementary material for: Multi‐Soliton Microcombs Enable Ultrafast Nanometric‐Precision Ranging and Photon‐Level Detection
Source: Adv Sci (Weinh). 2026 Jan 27;13(12):e16806. doi: 10.1002/advs.202516806 (PMC12948229; doi:10.1002/advs.202516806)
Supplement: Supplementary file 1 — Supporting File: advs74073‐sup‐0001‐SuppMat.docx. [file ADVS-13-e16806-s001.docx]

Supporting Information

Multi-soliton microcombs enable ultrafast nanometric-precision ranging and photon-level detection

*Jiawen Zhi, Xiaoyang Guo, Xusheng Yang, Brent E. Little, Sai T. Chu, Chenggang Shao, Mengyu Wang, Yan Liang, Peng Xie^*^, Weiqiang Wang^*^, and Hanzhong Wu^*^*

**Evaluation of power conversion efficiency at different microcomb states**

We have systematically characterized the optical power conversion efficiency of different microcomb states, as shown in **Figure S1**. The microcomb exhibits the high repetition rate of about 49  GHz, and the optical spectrum analyzer (OSA) used here provides the resolution of 6.16 GHz at 1560 nm, enabling clear resolution of individual comb lines. The power of each comb line is measured with the OSA, as illustrated in Figure S1A. In the recorded spectrum, the sum of the powers of other comb lines excluding the pump line is denoted as *P*_other_​, while the highest-power line corresponds to the pump power *P*_pump_​. The power conversion efficiency *η* is then calculated as, *η*=*P*_other_/(*P*_pump_+*P*_other_).


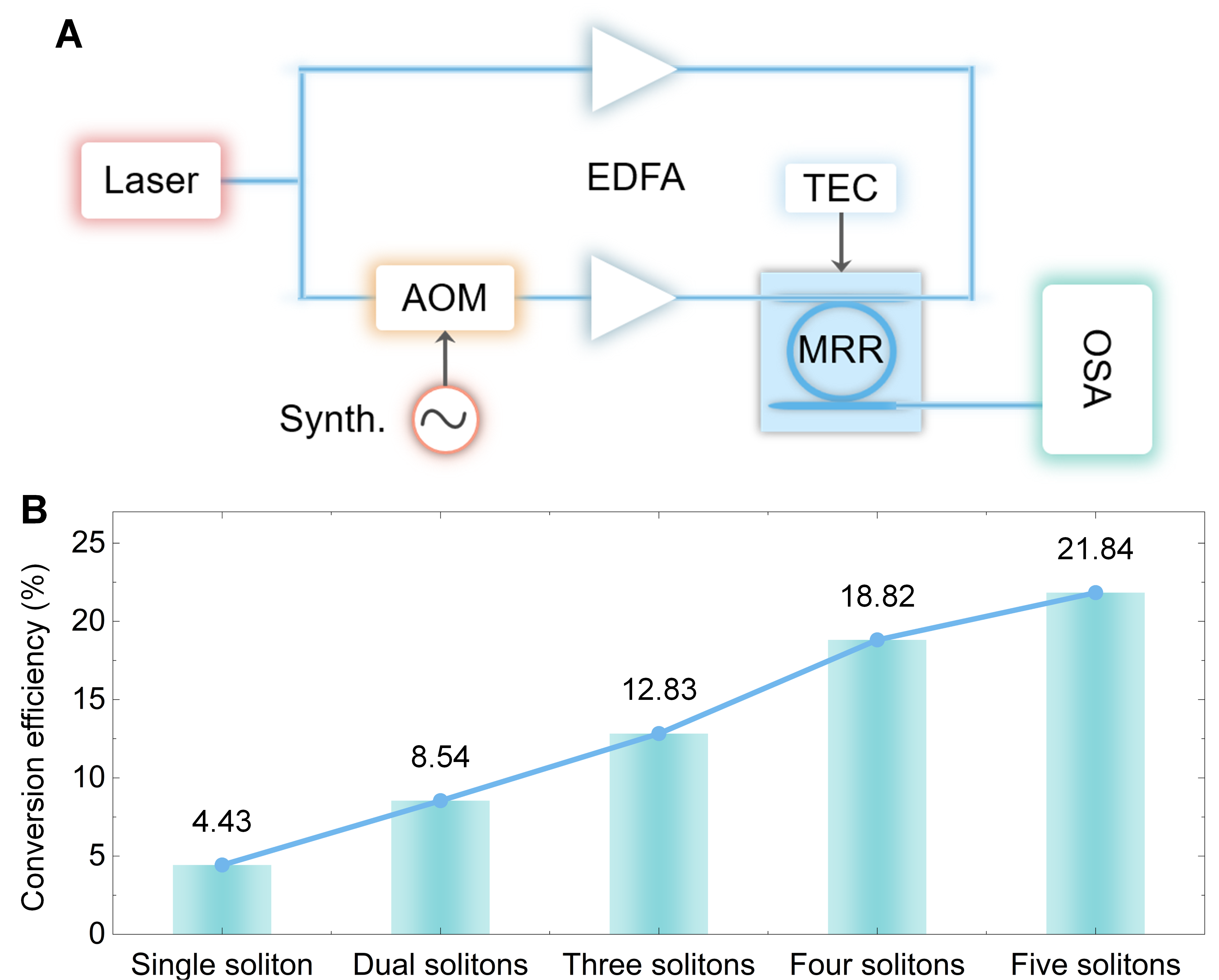


**Figure S1.** Evaluation of optical power conversion efficiency at different comb states. A) Measurement schematic of power conversion efficiency. AOM: acousto-optic modulator; Synth.: signal synthesizer; EDFA: Er-doped fiber amplifier; MRR: micro-ring resonator; TEC: temperature controller. B) Measurement results of power conversion efficiency.

The measured power conversion efficiencies for different soliton states are summarized in Figure  S1B. From the single-soliton to the five-soliton state, the power conversion efficiency increases nearly linearly. Specifically, the conversion efficiency is about 4.43% for the single-soliton state and reaches 21.84% for the five-soliton state. Experimental results demonstrate that employing multi-soliton states can effectively enhance the power conversion efficiency of the system. Furthermore, we evaluate the conversion efficiency for states with even higher soliton numbers, with values ranging between 22% and 28.5%. The departure from linear growth is attributed to the maximum power conversion capability of the microcomb itself.

**Characterization the intrinsic stability of the pulse interval**





**Figure S2.** Characterization results for intrinsic stability of pulse intervals. A) Spectrum of the dual-soliton microcomb. The inset is the experimental setup. B) Result of fast Fourier transform. C) Wrapped phase. D) Unwrapped phase, whose slope can be used to measure the distances. E) Measurement results for about 10000 s, based on the phase slope. F) Measurement results for about 10000 s, based on the single-wavelength phase. G) Allan deviations. The green line indicates the results from the phase slope, and the purple line corresponds to the results based on the single-wavelength phase.

We characterize the intrinsic stability of the pulse interval in the multi-soliton source by analyzing the two-soliton microcomb. Its optical spectrum is shown in **Figure S2**A, with the corresponding experimental setup illustrated in the inset. The microcomb output is recorded by an optical spectrum analyzer. The temporal separation between solitons is determined via dispersive interferometry.^[1]^ Figure S2B displays the result of applying the fast Fourier transform to the spectrum from Figure S2A. The peak is extracted and then inversely Fourier-transformed to obtain the wrapped phase shown in Figure S2C. After unwrapping, the linear phase profile in Figure S2D is obtained. The slope of unwrapped phase can be utilized to calculate the soliton interval *D* according to *D*=*dϕ*/*dω*, where *ϕ* is the unwrapped phase and *ω* is optical angular frequency. Furthermore, the soliton interval is finely measured via the phase of one single wavelength (here, 1549.997 nm) based on the results in Figure S2C.

We evaluate the stability over approximately 10000 s, with each individual measurement lasting 6 s. The results derived from the phase-slope method are presented in Figure S2E, yielding the standard deviation of 570 as. The corresponding results obtained from the single-wavelength method are shown in Figure S2F, with the standard deviation of 3.81 as. The Allan deviations are plotted in Figure S2G. For the phase-slope method, the precision reaches 384 as at 12 s, 175 as at 102 s, and 133 as at 1002 s averaging time. When using the single-wavelength approach, the precision achieves 2.57 as at 12 s, 1.15 as at 102 s, and 0.895 as at 1002 s averaging time. These results confirm that the soliton interval in the multi-soliton source maintains high stability. It should be noted that this level of soliton-interval stability does not affect the ranging performance, because the distance measurements rely on the phase difference between the measurement and reference interferograms, which effectively cancels out common-mode fluctuations.

**Extending the non-ambiguity range of distance measurement**

In the frequency comb based ranging, the non-ambiguity range is determined by half the pulse interval, which corresponds to *c*/(*n_g_*×*f_rep_*)/2. Here, *c* denotes the light speed in vacuum, *n_g_* is the group refractive index of air, and *f_rep_* is the repetition rate. Various methods have been developed to extend the non-ambiguity range, such as changing the repetition rate^[2]^ (i.e., the Vernier effect), pseudo-random phase modulation,^[3]^ intrinsic intensity modulation,^[4]^ and triple-comb interferometry.^[5]^ In this work, we demonstrate a simple approach inherently integrated within our system to expand the non-ambiguity range. The experimental setup is depicted in **Figure S3**A. Please note that, the local comb shown here does not participate in the non-ambiguity range extension. It is included merely to indicate that the configuration remains consistent with Figure 2A, requiring no significant modification in this step.

The comb laser is generated via the thermal-balance method employing two pump lasers. Both pumps originate from the same seed laser and are coupled into the microresonator in opposite directions (clockwise and counter-clockwise). The auxiliary laser is frequency-shifted by an acousto-optic modulator (AOM). Owing to the reflection effect within the microresonator, the output port contains a portion of the auxiliary laser, resulting in a stable heterodyne signal at the frequency equal to the AOM drive frequency (*f*_AOM_)​. Consequently, the system effectively operates as a phase-modulated LiDAR, where distance is derived from the phase of this heterodyne signal. The measured distance *L* is given by (*φ_meas_*-*φ_ref_*)/2*π*×*c*/(*n_p_*×*f*_AOM_)/2, with *φ_meas_* and *φ_ref_* representing the phases of the measurement and reference beams, respectively, and *n_p_* the phase refractive index of air. In our experiments, the AOM drive frequency is approximately 150 MHz for the signal comb and about 140 MHz for the local comb. The 150 MHz component is thus extracted using a bandpass filter.





**Figure S3.** Schematic and results of extending the non-ambiguity range. A) Ranging schematic using the beat frequency between the pump and auxiliary lasers. Cir.: circular; Col.: collimator; PD: photodetector; BPF: band-pass filter; BS: beam splitter. B) Spectrum of 151 MHz beat frequency with 63 dB signal-to-noise ratio. C) Spectrum of 152 MHz beat frequency with 63 dB signal-to-noise ratio. D) Allan deviations of the beat frequencies. E) Phase measurement by using the beat frequency. F) Results of the distance measurement by using the beat frequency.

Distance measurements are performed using two heterodyne frequencies, 151 MHz and 152 MHz, corresponding to non-ambiguity ranges of approximately 0.993 m and 0.987 m, respectively. A virtual synthetic wavelength is consequently generated, given by *c*/(152 MHz-151 MHz)≈300 m, which yields the non-ambiguity range of 150 m. Note that the solitons remain stable when the AOM drive frequency is tuned within a range of about 15 MHz. The spectra of the heterodyne signals are shown in Figures S3B and S3C, exhibiting the signal-to-noise ratio of 63 dB. The long-term stability is measured as 2.3×10^-12^ at 1 s and 1.4×10^-13^ at 128 s averaging time, as plotted in Figure S3D. The target distance is varied linearly in steps of 0.1 m, and the corresponding phase response also varies linearly, as presented in Figure S3E. When the displacement is larger (e.g., at 0.9 m position), the phase difference between the 151 MHz and 152 MHz signals becomes more obvious due to the Vernier effect. The measurement results are indicated in Figure S3F, where the midpoints represent the average of 100 measurements and the errorbars indicate the standard deviation. Within 1 m range, the measurement uncertainty remains below ±350 μm, which is smaller than 3 mm (i.e., *c*/49.001117GHz/2). This approach enables further distance measurements through dual multi-soliton interferometry.

**References**

[1] K.-N. Joo, S.-W. Kim, "Absolute distance measurement by dispersive interferometry using a femtosecond pulse laser," *Opt. Express* **2006**, *14*, 5954.

[2] H. Wu, T. Zhao, Z. Wang, et al., "Long distance measurement up to 1.2 km by electro-optic dual-comb interferometry," *Applied Physics Letters* **2017**, *111*, 251901.

[3] X. Guo, J. Zhi, H. Wu, "Pseudo-random phase modulation-assisted dual comb ranging with an extended non-ambiguity range," *Optics and Lasers in Engineering* **2025**, *189*, 108948.

[4] J. Fellinger, G. Winkler, P. E. C. Aldia, et al., "Simple approach for extending the ambiguity-free range of dual-comb ranging," *Opt. Lett.* **2021**, *46*, 3677.

[5] X. Zhao, X. Qu, F. Zhang, Y. Zhao, G. Tang, "Absolute distance measurement by multi-heterodyne interferometry using an electro-optic triple comb," *Opt. Lett.* **2018**, *43*, 807.
